# Non-invasive cardiac kinetic energy distribution: a new marker of heart failure with impaired ejection fraction (KINO-HF)

**DOI:** 10.3389/fcvm.2023.1096859

**Published:** 2023-05-02

**Authors:** Eva De Keyzer, Amin Hossein, Jeremy Rabineau, Marielle Morissens, Alexandre Almorad, Philippe van de Borne

**Affiliations:** ^1^ Department of Cardiology, Brugmann Hospital, Université Libre de Bruxelles, Brussels, Belgium; ^2^ Laboratoray of Physics and Physiology, Université Libre de Bruxelles, Brussels, Belgium; ^3^ Heart Rhythm Management Centre, European Reference Networks Guard-Heart, Universitair Ziekenhuis Brussel - Vrije Universiteit Brussel, Brussels, Belgium; ^4^ Department of Cardiology, Erasme Hospital, Université Libre de Bruxelles, Brussels, Belgium

**Keywords:** heart failure, reduced ejection fraction, e-health, kinocardiography, seismocardiography, ballistocardiography, point-of-care screening, aid-to-diagnosis

## Abstract

**Background:**

Heart failure (HF) remains a major cause of mortality, morbidity, and poor quality of life. 44% of HF patients present impaired left ventricular ejection fraction (LVEF). Kinocardiography (KCG) technology combines ballistocardiography (BCG) and seismocardiography (SCG). It estimates myocardial contraction and blood flow through the cardiac chambers and major vessels through a wearable device. Kino-HF sought to evaluate the potential of KCG to distinguish HF patients with impaired LVEF from a control group.

**Methods:**

Successive patients with HF and impaired LVEF (iLVEF group) were matched and compared to patients with normal LVEF ≥ 50% (control). A 60 s KCG acquisition followed cardiac ultrasound. The kinetic energy from KCG signals was computed in different phases of the cardiac cycle ($iK_{systolic};\;\Delta iK_{diastolic}$) as markers of cardiac mechanical function.

**Results:**

Thirty HF patients (67 [59; 71] years, 87% male) were matched with 30 controls (64.5 [49; 73] years, 87% male). SCG $\Delta iK_{diastolic}$, BCG $iK_{systolic}$, BCG $\Delta iK_{diastolic}$ were lower in HF than controls (*p* < 0.05), while SCG $iK_{systolic}$ was similar. Furthermore, a lower SCG $iK_{systolic}$ was associated with an increased mortality risk during follow-up.

**Conclusions:**

KINO-HF demonstrates that KCG can distinguish HF patients with impaired systolic function from a control group. These favorable results warrant further research on the diagnostic and prognostic capabilities of KCG in HF with impaired LVEF.

**Clinical Trial Registration**: NCT03157115.

## GRAPHICAL ABSTRACT

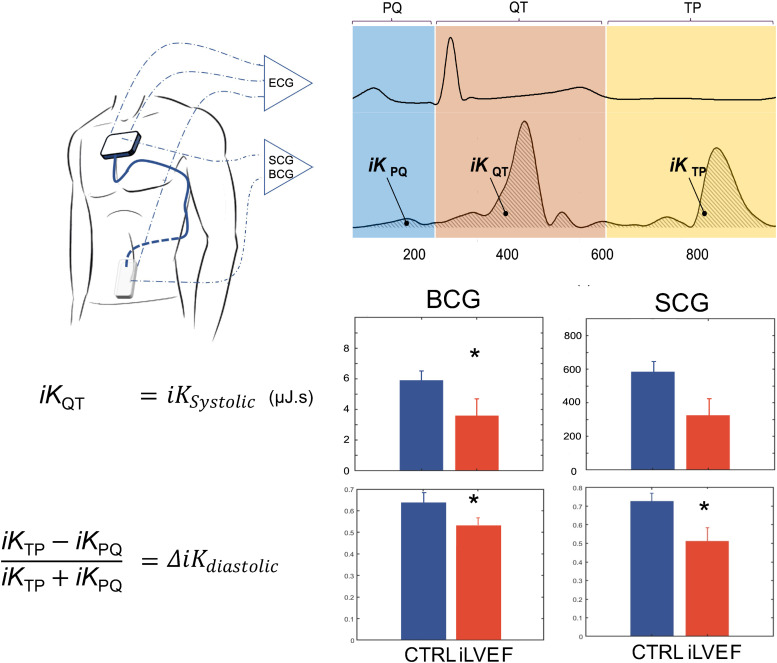

## 1. Introduction

Heart failure (HF) is a complex syndrome affecting an estimated 1%–2% of the population in developed countries. In the United States, it represents 6.5 million patients, accounting for ∼380.000 deaths and more than 3.5 million hospitalizations per year (1–3).

A major challenge in HF care is to prevent and shorten hospitalizations since they contribute significantly to the human and economic burden on patients and healthcare systems. The first few weeks after discharge entail an elevated mortality (4–6). Close clinical follow-up by general practitioners and cardiologists is needed but not always feasible given the limited resources in many geographical areas (7).

Home monitoring of patients has been investigated for years to intercept clinical or subclinical, indicators of increasing congestion. It aims to improve symptoms, prevent acute decompensation, and thereby the need for hospitalization for HF by enabling caregivers to modify treatment in a timely manner.

Recent years have witnessed an upsurge in the use of ballistocardiography (BCG) and seismocardiography (SCG), two techniques enabling the assessment of the inotropic state based on the measurement of body movements induced by cardiac contraction and blood flow in the cardiac chambers and major vessels (8, 9).

More recently, these techniques have been used in domains such as atrial fibrillation (10) and hypertension detection (11), heart failure monitoring (12, 13), cardiorespiratory fitness assessment (14, 15), and many others. Recent advances in the field of SCG were published by Taebi et al. (16).

Kinocardiography (KCG) is a subject-specific calibrated combination of linear and rotational SCG and BCG techniques. KCG is based on measures of 6 degrees-of-freedom (DOF) combining three-dimensional (3D) linear and 3D angular motion. These are recorded from sensors attached with electrodes to the presternal cutaneous surface (SCG) and the lumbar area (BCG).

In the present study, we used KCG metrics to measure kinetic energy distribution in different cardiac phases and compared them among subjects with impaired left ventricular ejection fraction (iLVEF) and a control group based on 2D-echocardiography.

## 2. Methods

### 2.1. Protocols and participants

All-comers between 20 and 85 years old at the echocardiography lab of the Brugmann University Hospital, Belgium, were invited to participate in the study. Exclusion criteria were pregnancy or any type of arrhythmia at the assessment time. In this feasibility study, patients with active right ventricular pacing were not included. Recordings were done in sinus rhythm only. This case-control study was performed between September 2017 and October 2021. The protocol complied with the Declaration of Helsinki and was approved by the local Ethics Committee (Brugmann University Hospital—CCB: B077201732405). The Belgian Federal Agency authorized the prototype device used in this clinical trial for Medicine and Health Products (FAMHP). Upon reception of the written informed consent, the participants' weight and height were measured. Then, they were equipped with the Kinocardiograph described in section 2.3 and were instructed to lie in a supine position on a bed for 5 min for stabilization. A blood-pressure measure was then performed (with Omron, EVOLV, HEM-7600T-E, Japan) after which a KCG recording was acquired for 60 s. Finally, participants were de-instrumented. A left ventricular ejection fraction (LVEF) below 50% was considered impaired.

### 2.2. Echocardiography

A basic comprehensive 2-dimensional transthoracic echocardiography was performed by a senior cardiologist who was blinded to the KCG measurements, with a Philips Epiq 7 ultrasound machine using a Philips X5-1 transducer (Eindhoven, The Netherlands).

The left ventricular outflow tract (LVOT) dimensions were obtained in the parasternal long-axis view during systole. The velocity time integral (VTI) at the LVOT was measured in the apical five-chamber view using pulsed-wave Doppler, and stroke volume (SV) was calculated using the following equation: SV = LVOT VTI × Cross Sectional Area of the LVOT (17). The left ventricular (LV) ejection fraction (LVEF) was measured with a modified Simpson's method (18). QRS duration was automatically measured for each of the 12-lead morphological analysis and expressed as ms, using the inbuilt software Extended Measurements Report of a Philips PageWriter TC50 electrocardiograph (Philips Medical Systems, Andover, MA, USA).

### 2.3. Kinocardiography data acquisition and analysis

**Figure 1:**
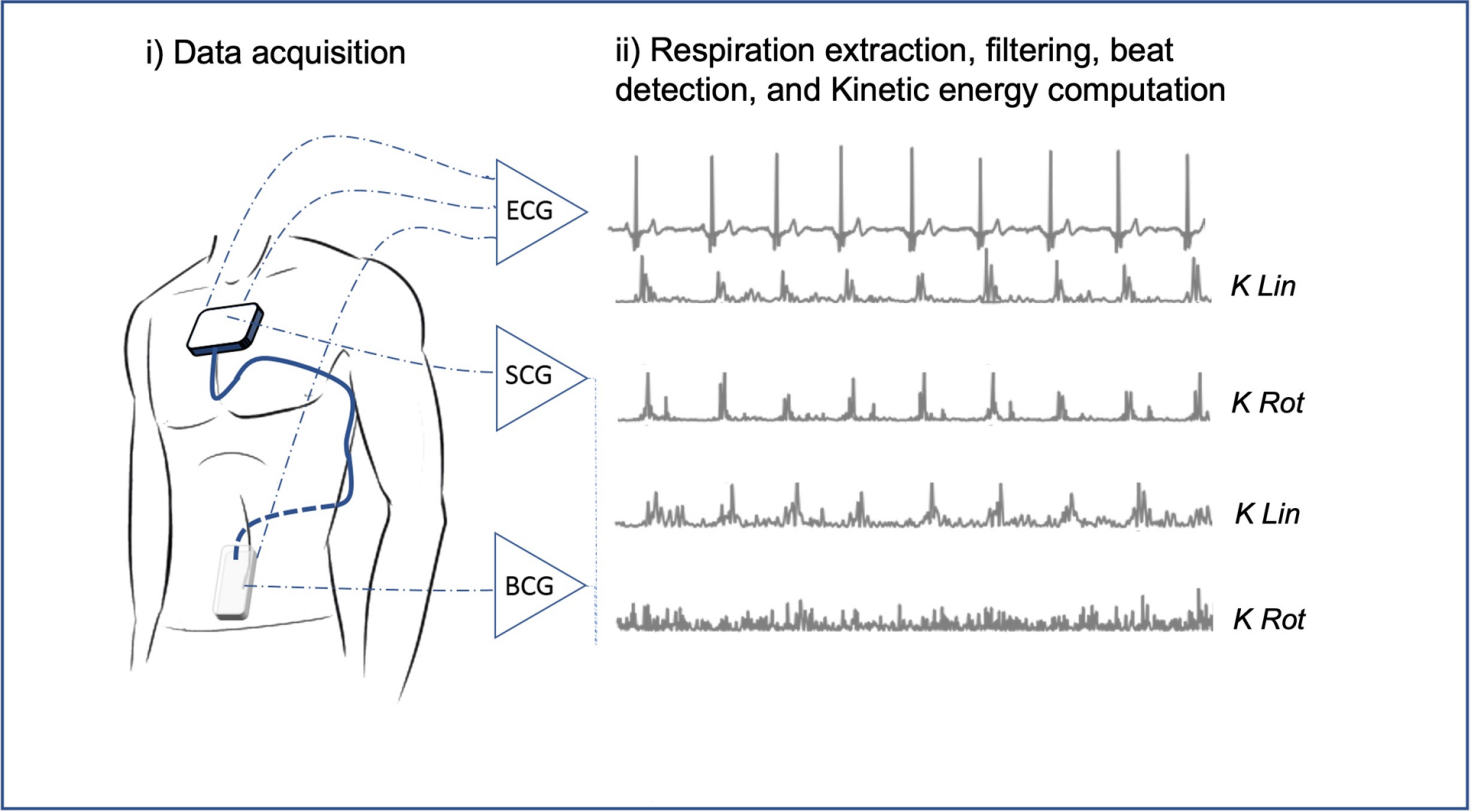
Kinocardiography signals acquisition and kinetic energy computation. From linear accelerations and angular rates raw acquisition on the chest (SCG) and in the lower back (BCG) to the kinetic energy metrics as described in section 2.4. SCG, seismocardiography; BCG, ballistocardiography; K, kinetic energy; Lin, linear; Rot, rotational.

The Kinocardiograph is a portable device with two embodiments, one of which (BCG) was placed over the lumbar region close to the subject's center of mass and the other one (SCG) over the manubrium sterni (Figure 1). The device records a two-lead ECG at 200 Hz together with 3D linear (Lin) accelerations and 3-DOF rotational (Rot) angular velocities from the sternum and the lumbar region as described in previous publications (19, 20). Ensemble averaging (EA) of signals was performed on all heartbeats. The time reference for the EA is a fixed interval that considers the cardiac activity preceding atrial depolarisation occurring before the R peak. Indeed, the beginning of the n-th interval started before the P wave, at time $k_{n,start}=\;R_{n}-\;\Delta$, where $R_{n}$ is the time of the n-th R peak and Δ is 200 ms. The end of the cardiac cycle of interest was assumed to be $k_{n,end}=\;R_{n}+\;\max[RR_{i}]$ where $RR_{i}$ represent all the RR intervals of the current record. The mean was taken on all the beats to obtain an average ECG signal. Based on this, an EA was calculated for each channel of the BCG and SCG recordings.

**Figure 2:**
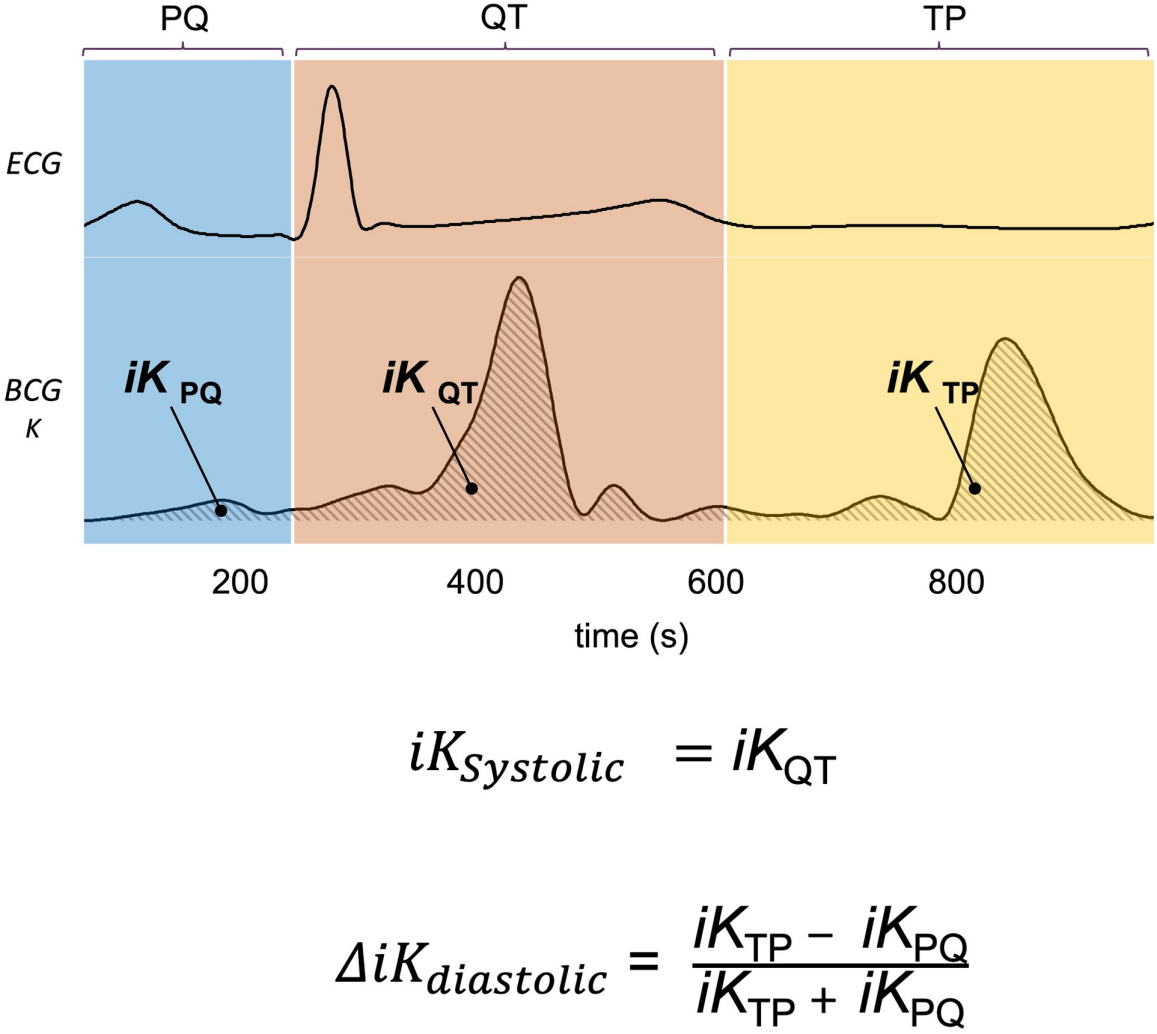
Kinocardiography metrics computation. Illustration of the PQ, QT, and TP phases segmented on the ECG and displayed for BCG kinetic energy (K). The time integrals of K are computed on each of these phases providing $iK_{PQ}$, $iK_{QT}$, and $iK_{TP}$ respectively. Based on these, $iK_{systolic}$ and $\Delta iK_{diastolic}$ are computed as displayed on the figure and further described in section 2.4.

Based on these acquisitions, the time integrals of kinetic energy (BCG $iK_{Lin}$, $iK_{Rot}$ and SCG i $K_{Lin}$, $iK_{Rot}$) were computed in different phases of the cardiac cycle as described in a previous publication (20) and summarized in Figure 2. The three cardiac cycle phases were: from the start of the P wave to the start of the QRS complex (PQ phase, late diastole), from the start of the QRS complex to the end of T wave (QT phase, systole), and from the end of the T wave to the start of the next *P* wave (TP phase, early diastole). These cardiac phases are illustrated in Figure 2.

These metrics have shown to be reproducible (21). Differences in *iK* metrics have been associated with differences in SV, LVEF, and cardiac output (CO) in healthy subjects during a dobutamine-induced hemodynamic increase (19, 22), with an increase during voluntary apnea (23) and obstructive apnea (24), and with a surge during sympathetic activation (25).

In this work, the following metrics to measure systolic and diastolic impairment were used (Figure 2):

1) The iK during the systolic phase was computed as: $iK_{systolic}=iK_{QT}$ (3)

This led to the metrics SCG $iK_{systolic}$ and BCG $iK_{systolic}$ for SCG and BCG, respectively. We hypothesize that these metrics reflect the contractility of the left ventricle.

2) The difference of *iK* between the TP and PQ phases (passive and active filling, respectively) was normalized by *iK* during the complete diastolic phase (TQ = TP + PQ): $\;\Delta iK_{diastolic}=\frac{iK_{TP}-iK_{PQ}}{iK_{TP}+iK_{PQ}}$ (4)

We thus obtained the metrics SCG $\Delta iK_{diastolic}$ and BCG $\Delta iK_{diastolic}$ for SCG and BCG, respectively. We hypothesized that these metrics reflect the difference of kinetic energy exerted between the passive and active diastolic phases, normalized by the kinetic energy generated throughout the entire ventricular diastole.

### 2.4. Statistics

All data analyses were performed offline using a proprietary software toolbox under Matlab (Mathworks Inc.®).

Data are presented as median [Q1; Q3]. The characteristics of each group were compared by a two-sample t-test in case of normal distribution or a Wilcoxon signed rank test in case of non-normal distribution. A Lilliefors test was used to test if the difference between sample populations compared was normally distributed. At study closure, an exploratory *post-hoc* analysis was performed to assess whether KCG metrics have predictability characteristics in the group with iLVEF. A Cox proportional hazards approach was used to assess univariate and multivariate associations with survivability. When several parameters showed significance, they were included in a single model and compared to the initial models with a log-likelihood ratio and a Chi-square distribution with a degree of freedom equal to the number of predictor variables being assessed.

The survival was estimated by the Kaplan–Meier method, with the follow-up period starting at the index echocardiogram until study closure, and group differences assessed with the log-rank test. For each parameter, the cut-off was set as the mean value between the group without and with adverse events. A *p*-value less than 0.05 was considered significant to compute 95% confidence intervals.

### 2.5. Estimation of a score and matching

Patients with reduced (rLVEF; LVEF ≤ 40%) or mildly reduced (mrLVEF; LVEF 41%–49%) LVEF were classified based solely on echocardiography parameters according to the 2021 ESC Guidelines (26). rLVEF and mrLVEF were labeled together as iLVEF (iLVEF group). All the other patients with normal LVEF (≥50%) were considered as controls. A matching score was computed to select an equal number of control patients to match the HF group. The matching score was based on age, sex, BMI, and blood pressure. Each patient from the iLVEF group was matched to a patient from the control group with the best matching score possible.

## 3. Results

This study included 131 patients. 5 patients were excluded for technical issues. In total, 126 patients (63 [49; 71] years old, 54% male) were kept for analyses. 96 patients had a normal LVEF and 30 an impaired LVEF (11 mrLVEF and 19 rLVEF). 30 patients from the group with normal LVEF and the highest matching score based on age, sex, BMI, and blood pressure when compared to the HF group were selected as the matched control group. Clinical characteristics, echocardiographic and KCG parameters, HR, and blood pressure of the iLVEF group, and the matched control group are presented in Table 1.

**Table 1:** Baseline patient characteristics after score matching.

	HF group	Matched Control	*p*-value
Number (*n*)	30	30	-
Gender (% male)	87	87	1
Age (years)	67.0 [59.0; 71.0]	64.5 [49.0; 73.0]	0.34
BMI (kg/m^2^)	25.4 [23.1; 30.9]	27.6 [25.2; 33.3]	0.1
Number of heartbeats	67.5 [60.0; 82.0]	65.0 [58.8; 74.0]	0.1
LVEF (%)	34.0 [27.8; 42.6]	61.0 [54.0; 65.0]	**0.0001**
Complete BBB (%)	30	7	**0.01**
Left BBB (*n*)	8	1	-
Right BBB (*n*)	1	1	-
QRS width (ms)	104 [93.0; 127.0]	94.0 [87.5; 102.0]	**0.02**
With BBB (ms)	154.0 [126.0; 160.5]	139.0 [139.0; 139.0]	-
QTc duration (ms)	411.7 [381.7; 433.2]	375.3 [346; 397.4]	**0.01**
IVSd (mm)	10 [8.5;12]	11 [9;12]	0.9
LVIDd (mm)	59 [52;66]	49 [45;55]	0.001
Chronic kidney disease (%)	23	7	1
Stroke (%)	13	7	0.4
COPD (%)	17	3	0.09
History of arrhythmia (%)	53	17	**0.003**
Valvular disease (%)	7	7	1
Coronary artery disease (%)	53	20	**0.008**
Smoker (%)	23	13	0.3
Dyslipidemia (%)	63	50	0.3
Arterial hypertension (%)	60	77	0.17
Diabetes (%)	37	33	0.8
Medications			
Beta blockers (%)	83	50	**0.007**
SGLT2i (%)	0	0	1
ACEi (%)	77	50	**0.03**
ARB (%)	10	10	1
ARNi (%)	27	3	**0.01**
MRA (%)	13	7	0.4
Calcium antagonist (%)	17	37	0.08
VKA (%)	17	3	0.09
Echocardiography			
Heart rate (bpm)	68 [60; 79]	62 [60; 73]	0.3
E/A	0.83 [0.56; 1.4]	1.0 [0.7; 1.2]	0.6
E (m/s)	0.64 [0.50; 0.80]	0.65 [0.50; 0.84]	0.6
A (m/s)	0.71 [0.56; 0.93]	0.73 [0.63; 0.87]	0.6
E/e’	11 [8.5; 13]	9.3 [6.1; 12]	0.008
e’ lateral (cm/s)	6.5 [4.5; 8.6]	9.5 [8.2; 12.0]	0.01
e’ median (cm/s)	5.2 [4.0; 5.8]	6.7 [5.4; 9.2]	0.01
SV indexed (ml/m^2^)	35 [27; 41]	31 [29; 37]	0.3
TR vmax (m/s)	2.6 [2.3; 2.7]	2.6 [2.4; 3.0]	1
LA diam (mm)	40 [36; 44]	38 [35; 44]	0.7
Blood pressure			
Systolic (mmHg)	126 [109; 139]	136 [117; 146]	**0.001**
Diastolic (mmHg)	70 [64; 81]	80 [70; 82]	**0.001**
Kinocardiography			
SCG $iK_{systolic}$ (µJ.s)	190 [65; 880]	570 [80; 1,000]	0.05
SCG $\Delta iK_{diastolic}$	0.60 [0.31; 0.79]	0.73 [0.70; 0.82]	**0.01**
SCG $iK_{PQ/TQ}$	0.20 [0.11; 0.35]	0.14 [0.09; 0.15]	**0.01**
SCG $iK_{TP/TQ}$	0.80 [0.65; 0.89]	0.86 [0.85; 0.91]	**0.01**
BCG $iK_{systolic}$ (µJ.s)	3.60 [1.70; 8.90]	5.80 [1.40; 15.0]	**0.03**
BCG $\Delta iK_{diastolic}$	0.54 [0.35; 0.71]	0.66 [0.59; 0.81]	**0.02**
BCG $iK_{PQ/TQ}$	0.23 [0.14; 0.32]	0.17 [0.09; 0.21]	**0.03**
BCG $iK_{TP/TQ}$	0.77 [0.68; 0.86]	0.83 [0.79; 0.90]	**0.03**

Values are expressed median [Q1; Q3].

BMI, body mass index; bpm, beat per minute; number of heartbeats, number of heartbeats used to compute KCG metrics; LVEF, left ventricle ejection fraction; BBB, bundle branch block; QTc, QT interval corrected; IVSd, interventricular septal end diastole; LVIDd, left ventricular internal diameter end diastole; COPD, chronic obstructive pulmonary disease; SGLT2i, SGLT2 inhibitor; ACEi, angiotensin-converting enzyme inhibitor; VKA, vitamin K antagonist; SV, stroke volume; TR vmax, tricuspid regurgitation maximum velocity; LA diam, left atrial diameter.

*p*-value below 0.05 were put in bold.

As presented in Table 1, heart rate, interventricular septal thickness in end diastole (IVSd), left atrial diameter, indexed SV, E/A, and maximum velocity of tricuspid regurgitation were comparable between groups. iLVEF patients had lower systolic and diastolic blood pressure (126 [109; 139] and 70 [64; 81] vs. 136 [117; 146] and 80 [70; 82] mmHg, respectively, *p* < 0.001). Furthermore, the QRS width was significantly larger in the iLVEF group (104.0 [93.0;127.0] vs. 94.0 [87.5;102.0] ms, *p* = 0.02) with more complete bundle branch block (BBB) morphology (30% vs. 7%, *p* = 0.01). The QTc duration was also longer in the iLVEF group (411.7 [381.7; 433.2] vs. 375.3 [346; 397.4] ms, *p* < 0.01). The left ventricular internal diameter at end diastole (LVIDd) was larger in the iLVEF group (59 [52; 66] vs. 49 [45; 55] mm, *p* < 0.01), and their diastolic parameter E/e' was higher (11 [8.5;13] vs. 9.3 [6.1;12], *p* < 0.01). A higher proportion of iLVEF was treated with beta-blockers and ACEi (83% vs. 50%, 77% vs. 50%, *p* < 0.01 and *p* < 0.05, respectively). Also, the iLVEF group patients had a higher proportion of history of arrhythmia and coronary artery disease (53% vs. 17% and 53% vs. 20%, respectively, *p* < 0.01).

Baseline patient characteristics within HF group are presented in Supplementary Material.

### 3.1. KCG group comparison

**Figure 3:**
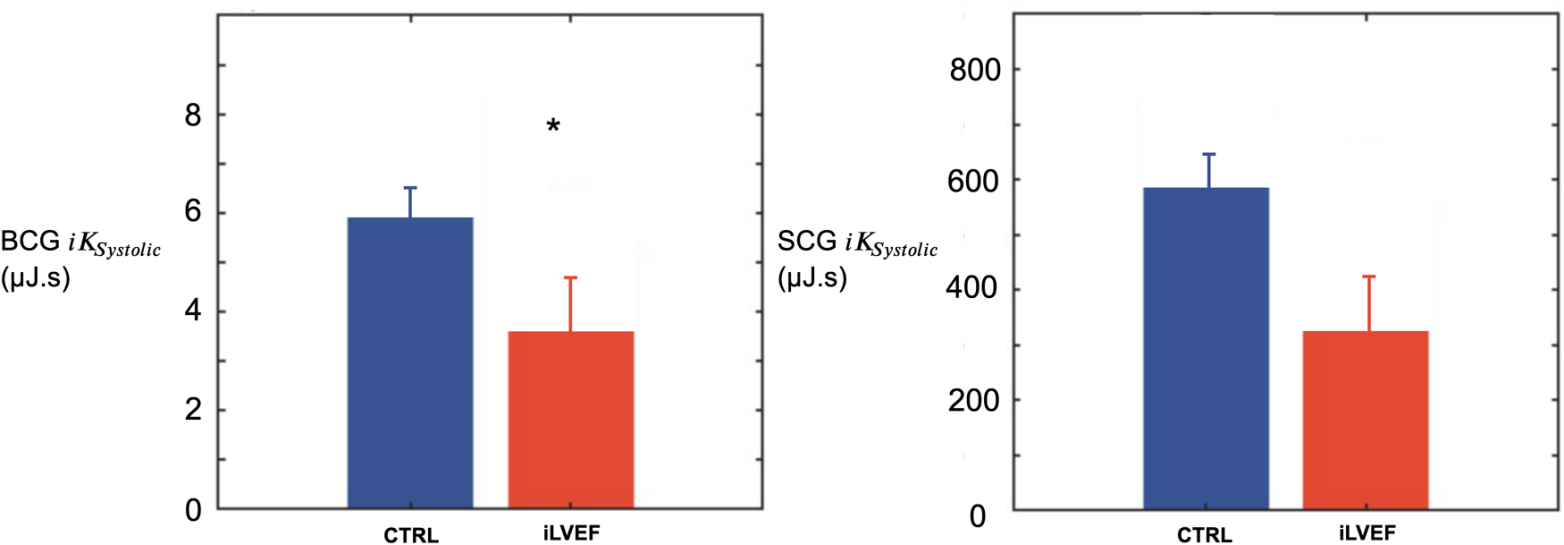
Systolic kinetic energy results. Systolic kinetic energy (mean and standard error of the mean) for BCG and SCG among iLVEF patients and matched controls patients with impaired left ventricular ejection fraction (*: <0.01). CTRL, control; iLVEF, patients with impaired left ventricular ejection fraction.

The BCG $iK_{systolic}$ was lower in iLVEF compared to the matched control group (3.6 [1.7; 8.9] µJ.s vs. 5.8 [1.4; 15.0] µJ.s, respectively *p* < 0.03, Figure 3). The SCG $iK_{systolic}$ did not differ between iLVEF and the matched control group (190 [65; 880] µJ.s and 570 [80; 1,000] µJ.s, respectively, Figure 3).

**Figure 4:**
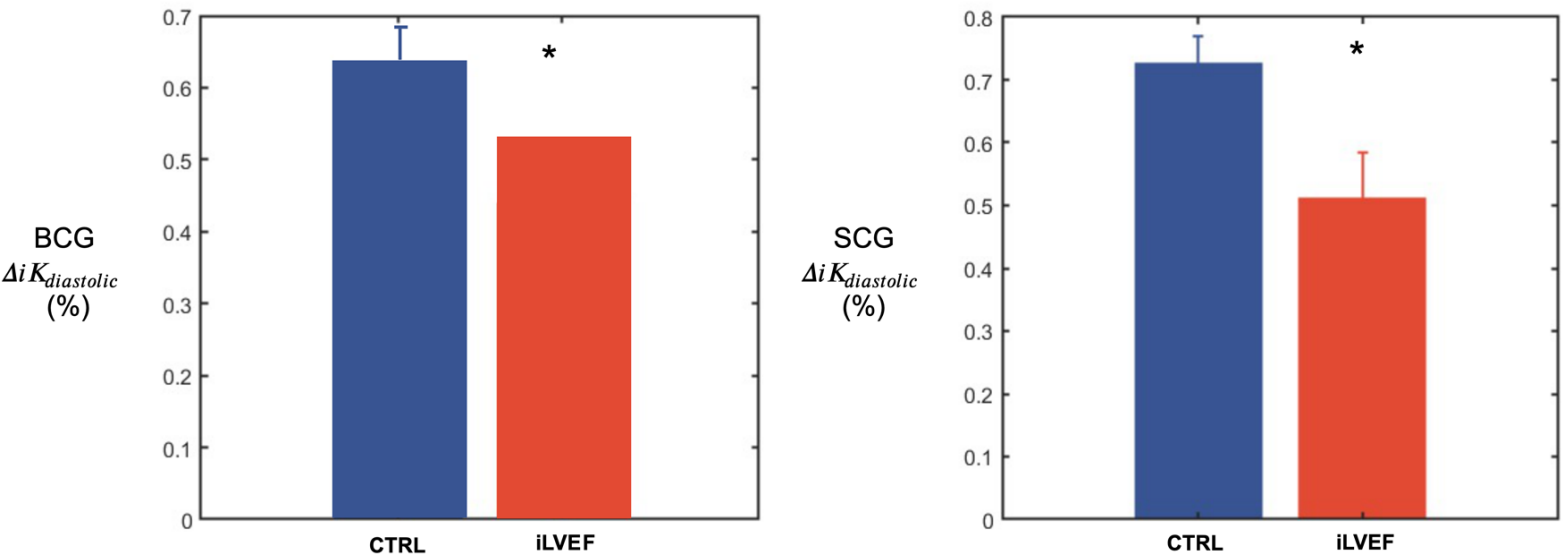
Diastolic kinetic energy results. Diastolic kinetic energy (mean and standard error of the mean) for BCG and SCG among matched controls and iLVEF patients with impaired left ventricular ejection fraction (*: <0.01). CTRL, control; iLVEF, patients with impaired left ventricular ejection fraction.

$\Delta iK_{diastolic}$ was found to be lower in the iLVEF group than in the matched control group for both BCG (0.54 [0.35; 0.71] and 0.66 [0.59; 0.81], respectively, *p* < 0.02, Figure 4) and SCG (0.60 [0.31; 0.79] and 0.73 [0.70; 0.82], respectively, *p* < 0.01, Figure 4).

### 3.2. Predictors of patients' survival

**Figure 5:**
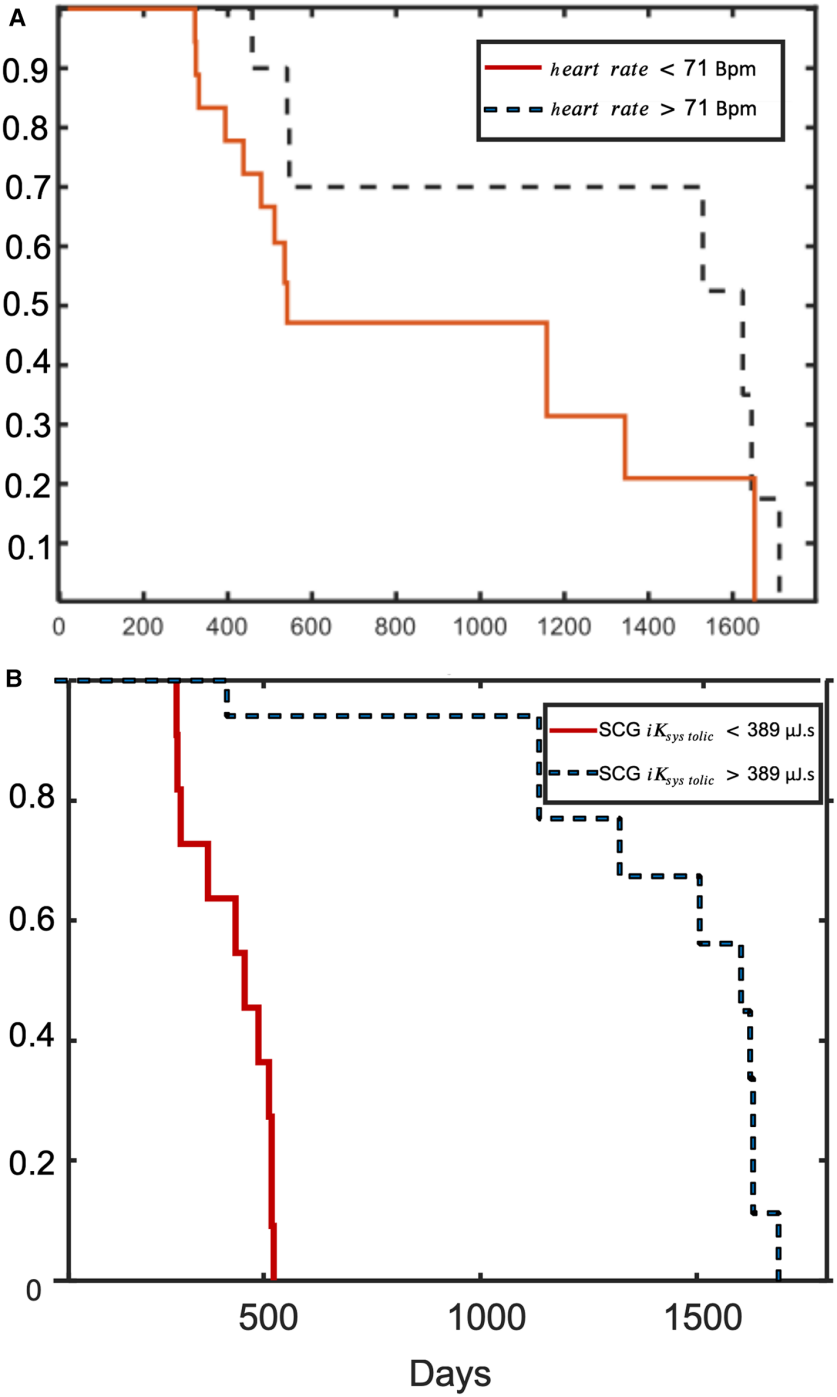
Kaplan–Meier probability of survival. Kaplan–Meier curves of event-free survival according to (**A**) SCG $iK_{systolic}$; and (**B**) heart rate.

During a total follow-up of 5 years after first inclusion, 21 participants of the iLVEF group were still alive. Based on multivariate analysis (Table 2), the proportion of patients with iLVEF surviving 4 years was significantly lower in patients with SCG $iK_{systolic}$ <390 µJ.s compared to SCG $iK_{systolic}$ >390 µJ.s (log rank *p* = 0.001) (Figure 5A, Table 2). There was also an observed difference in survival between iLVEF patients with a heart rate >71 bpm and those with a heart rate <71 bpm (log rank *p* = 0.04, Figure 5B, Table 2). A Cox multivariate model including both SCG $iK_{systolic}$ and heart rate was generated and showed no significant improvement, as compared to the model including only SCG $iK_{systolic}$ (*p* = 0.3). Age, LVEF, chronic kidney disease, and BBB were not significant predictors of survivability in the tested group.

**Table 2:** Multivariate Cox proportional hazards model, continuous variables.

Population (*n* = 30: 21 alive-unknown, 9 deaths)			
Parameter	HR	95% CI	*p*-value
Gender	−0.17	−0.58, 0.24	0.8
Age	−0.0300	−0.0310, −0.0297	0.2
BMI	−0.021	−0.024, −0.019	0.7
LVEF	0.019	0.018, 0.020	0.5
Complete Bundle Branch Block	−0.277	−0.459, −0.095	0.5
Chronic kidney disease	0.25	−0.03, 0.53	0.6
SCG $iK_{systolic}$	0.91	0.83, 0.99	**0** **.** **0013**
SCG $\Delta iK_{diastolic}$	1.2	0.5, 1.9	0.15
BCG $iK_{systolic}$	−314	-	0.1
BCG $\Delta iK_{diastolic}$	0.91	0.2, 1.6	0.27
Heart rate	−0.050	−0.051, −0.050	**0** **.** **04**
E/A	−0.36	−0.42, −0.31	0.12
E/e’	−4.72	−35.8994, 26.4636	0.4
Systolic Blood pressure	0.005	0.004, 0.005	0.6

Cox regression analysis for assessing the association between variables and survival rate. Among the tested parameters, only SCG $iK_{systolic}$ and hearth rate have shown to be significant predictor of survivability in the tested group. Having a decreased SCG $iK_{systolic}$ increased the mortality risk by 2.5, while having an increased hearth rhythm increased the mortality risk by 1.05.

*p*-value below 0.05 were put in bold.

## 4. Discussion

In the KINO-HF exploratory study, the comparison of KCG metrics between patients with impaired LVEF and matched control subjects reveals that the iLVEF group is associated with lower values of systolic kinetic energy ($iK_{systolic}$) and diastolic kinetic energy ($\Delta iK_{diastolic}$). Also, in iLVEF patients, higher values of $iK_{systolic}$ were associated with a better survival.

### 4.1. Kinocardiography metrics rationale

Recent studies have shown interest in using intra-ventricular kinetic energy through 4D flow MRI to assess left and right ventricular functions (27, 28). These have shown very promising results allowing a better understanding of the impact of cardiac disease on intra-cardiac hemodynamics but also opening the perspective of new ways to characterize HF.

The aim of KCG is also to compute a kinetic energy but through body surface accelerations and angular rates in a simpler and more indirect way when compared to cardiac MRI derived kinetic energy (MRI KE). In particular, studies have shown that LV systolic MRI KE decreased significantly when comparing controls to patients with myocardial infarction with decreased EF (29, 30). Moreover, others have shown that most flow components of the late (A) diastolic MRI KE increased in HF patients with reduced LVEF in comparison to healthy subjects (31). Inspired by these results, this study introduces two metrics based on KCG: (1) systolic kinetic energy, named $iK_{systolic}$, analog to LV systolic MRI KE and (2) diastolic gradient kinetic energy, named $\Delta iK_{diastolic}$, reflecting the E and A MRI KE relative distribution.

### 4.2. Systolic kinetic energy

BCG $iK_{systolic}$ was significantly lower in patients from the iLVEF group, reflecting the reduced amount of force developed by the ventricles in these patients. This is in line with the reduction of LV systolic MRI KE as detected by cardiac MRI in patients with impaired LVEF (31), or when comparing controls to patients with myocardial infarction with decreased EF in experimental and clinical settings (29, 30, 32). However, in this study, HF patients and matched control showed comparable resting indexed SV (Table 1). Therefore, the correlation between BCG $iK_{systolic}$ and SV found in a previous study in healthy participants during a dobutamine-induced increase in contractility (19) is not extensible to pathological cases. The correlation between BCG $iK_{systolic}$ and SV might mostly be accurate for intra-patient follow-up, as described in two studies: (1) during a cardiac deconditioning measured by cardiac MRI due to long-duration head-down tilt bed rest, where a significant decrease of this metric was shown (33); and (2) in a recent study showing that this metric followed significantly the change in the cardiac hemodynamic load in HF patients (34).

Interestingly, a study showed the high sensitivity of BCG $iK_{systolic}$ to beta adrenergic stimulation (19), therefore the higher proportion of beta blockers in the HF group compared to the matched control group might have contributed to the decrease in BCG $iK_{systolic}$ in this study.

The same was not observed for SCG $iK_{systolic}$. This might be explained by the discrepancy in the proportion of patients with ventricular dilatation: 41% (iLVEF) vs. 7% (control group) had a LVIDd larger than 60 mm (*p* < 0.01, Table 1). In our cohort, $iK_{systolic}$ SCG was found to be much higher in patients with ventricular dilatation than in the other patients with impaired LVEF (380 [80; 980] µJ.s vs. 84 [55; 210] µJ.s, *p* < 0.0001, Table 3). Since SCG measures a larger cardiac mass displacement in patients with dilated left ventricle, this confounding factor might have increased the SCG $iK_{systolic}$ values in the iLVEF group leading to values comparable to the control group.

**Table 3:** KCG parameters from HF group with LVIDd inferior or superior to 60 mm.

	With LVIDd ≤60 mm (*n* = 18)	With LVIDd >60 mm (*n* = 12)	*p*-value
SCG $iK_{systolic}$ (µJ.s)	84 [55; 210]	380 [80; 980]	0.0001
SCG $\Delta iK_{diastolic}$	0.50 [0.24; 0.74]	0.74 [0.45; 0.82]	0.5
BCG $iK_{systolic}$ (µJ.s)	2.2 [0.6; 3.9]	10 [3.9; 36]	0.01
BCG $\Delta iK_{diastolic}$	0.48 [0.34; 0.62]	0.62 [0.47; 0.78]	0.3

Values are expressed in median [Q1; Q3]. LVIDd, left ventricular internal diameter end diastole.

Interestingly, BCG $iK_{systolic}$ was also found to be higher in patients with ventricular dilatation than in the other iLVEF patients (10 [3.9; 36] µJ.s vs. 2.2 [0.6; 3.9] µJ.s, *p* < 0.01, Table 3). As such, the ventricular dilation can impede the expected decrease of SCG $iK_{systolic}$ in the iLVEF group but not the decrease of $iK_{systolic}$ BCG. The essence of BCG is to measure micromovements of the body in reaction to blood flow through the vasculature, mainly the aorta, making this metric less sensitive to mechanical LV motion and LV dilatation (35).

Furthermore, in this study, a decreased SCG $iK_{systolic}$ was shown to be a significant predictor of all-cause mortality among patients with impaired LVEF. Similarly, on datasets of nearly the same size, others have shown that some features of the SCG signal can be extracted to predict HF readmission, one of which is the SCG amplitudes (36). However, our result seems to conflict with the elevated values of high $iK_{systolic}$ in patients with dilated ventricles, who are known to have a worse prognosis if associated with HFrEF. This emphasizes that this result was obtained in a relatively small population with varying LV morphology within the iLVEF group, and larger studies are needed to be able to conclude the predictability of $iK_{systolic}$.

### 4.3. Diastolic kinetic energy

For both BCG and SCG, $\Delta iK_{diastolic}$ was found to be significantly decreased in the iLVEF group when compared to the matched control. In contrast, diastolic function parameters such as E/A, or tricuspid regurgitation maximum velocity were equally distributed in both groups. However, as expected, E/e' was higher in the iLVEF group (11 [8.5;13] % vs. 9.3 [6.1;12] %, *p* < 0.01). $\Delta iK_{diastolic}$ sought to reflect energy distribution along the diastole (passive and active filling). MRI studies have shown that HF patients with impaired LV function are characterized by altered diastolic flow routes through the LV and impaired preservation of MRI KE during late diastole (31). In this phase of the heart cycle corresponding to atrial systole, Eriksson et al. further described high MRI KE in HF patients reflecting impaired active relaxation of the myocardium caused by a less compliant myocardium (31). This is in line with our results, where we report a significant increase in the difference of energy developed by the heart during the diastole ($\Delta iK_{diastolic}$).

The differences observed by KCG in the diastolic parameters between iLVEF and control patients might be partly due to reduced LV compliance in patients with impaired LV function. Furthermore, we decomposed the gradient in its early ($iK_{TP/TQ}$) and late ($iK_{PQ/TQ}$) components normalized by the total diastolic kinetic energy (Table 1). The early diastole energy decreased while the late diastole energy increased in the iLVEF group and thus leading to a lower diastolic gradient ($\Delta iK_{diastolic}$). This aligns with the increase of intracardiac filling pressures during the late diastole in the iLVEF group.

### 4.4. Limitations

The present work presents some limitations worth noting. The definitions of the systolic and diastolic kinetic energy parameters are inherently an approximation based on ECG detection. Furthermore, we expect a slight delay of the systolic and diastolic events acquired for the BCG energies compared to the ECG segmentation. Moreover, the possibility of using KCG in specific clinical scenarios, such as atrial fibrillation or intracardiac devices, is still to be investigated. Indeed, patients with pacemaker were excluded from this study to avoid the uncertainties of ventricular asynchrony induced by right ventricular stimulation on KCG. However, they should not be excluded in future studies.

The control population was well matched with respect to general characteristics of population such as gender, BMI, and age but presented different characteristics regarding medical treatment and cardiac pathologies such as BBB. However, the control patients were recruited at the cardiology department, and therefore should represent a relevant sample of the population that would need to be discriminated and ruled out of a HF diagnosis. Eligible patients with left BBB recruited in this study were later implanted with a cardiac resynchronization device. The fact that larger LVIDd among iLVEF patients increased $iK_{systolic}$ contrasts with the results showing that increased $iK_{systolic}$ predicts poor prognosis. This calls for larger cohorts to be studied to better understand of the effect of HF etiology on the $iK_{systolic}$ metric.

In this study, both, $iK_{systolic}$ and $\Delta iK_{diastolic}$ have shown group differences when comparing the group with iLVEF to the matched control group. However, the metrics values overlap between both groups, and it is therefore not possible to define a single cut-off value achieving good discrimination between iLVEF and controls. A joint parameter combining BCG and SCG metrics could be developed to achieve this. To a further extent, machine learning algorithms combining more than two metrics could be developed on larger datasets and might provide accurate discrimination between these two groups. Further studies are needed to confirm this potential.

We acknowledge that LVEF is simply one parameter of systolic function among others, and that a normal LVEF does not exclude systolic dysfunction, as in cardiac amyloidosis or when considering the more subtle systolic dysfunction seen in HF patients with preserved ejection fraction.

## 5. Conclusion

This study demonstrates that patients with iLVEF are associated with lower values of $iK_{systolic}$ and $\Delta iK_{diastolic}$ when compared to a matched control group. In addition, in iLVEF patients, $iK_{systolic}$ seems to be an independent predictor of survivability. Warranting further studies to evaluate KCG accuracy in different clinical settings and in a blind setup, these scalar metrics might be useful for screening for impaired LVEF.

## Data Availability

The original contributions presented in the study are included in the article/**Supplementary Materials**, further inquiries can be directed to the corresponding author/s.
