# Supplementary material for: Non-invasive cardiac kinetic energy distribution: a new marker of heart failure with impaired ejection fraction (KINO-HF)
Source: Front Cardiovasc Med. 2023 May 2;10:1096859. doi: 10.3389/fcvm.2023.1096859 (PMC10185762; doi:10.3389/fcvm.2023.1096859)
Supplement: Supplementary file 1 [file Table1.pdf]

Supplementary Material

Table A1**: Baseline patient characteristics within HF group**

	**HFrEF**	**HFmEF**
**Number (n)**	19	11
**Gender (% Male)**	95	73
**Age (years)**	67.0 [59.2; 70.5]	65.0 [59.2; 72.2]
**BMI (kg/m^2)**	25.1 [22.9; 31.1]	25.9 [23.4; 30.6]
**LVEF (%)**	29.0 [25.0; 33.0]	43.0 [41.2; 45.8]
**Complete BBB (%)**	37	27
**Left BBB (n)**	5	3
**Right BBB (n)**	1	0
**QRS width (ms)**	109.0 [96.0; 154.5]	104.0 [84.0; 117.0]
**With BBB (ms)**	155.0 [133.8; 161.5]	126.0[120.0; 132.0]
**>120 ms (n)**	7	1
**QTc duration (ms)**	411.7 [381.7; 433.2]	375.3 [346; 397.4]
**IVSD (mm)**	9.5 [6; 12]	11 [9.5; 13]
**LVEDD (mm)**	62 [55; 69]	51 [44; 57]
**Chronic kidney disease (%)**	16	36

**Stroke (%)**	5	27
**COPD (%)**	16	18
**History of arrhythmia (%)**	58	45
**Valvular Disease (%)**	11	0
**Coronary artery disease (%)**	74	18
**Smoker (%)**	21	27
**Dyslipidemia (%)**	58	73
**Arterial Hypertension (%)**	63	55
**Diabetes (%)**	37	36
**Medications**		
**Beta blockers (%)**	84	82
**SGLT2i (%)**	0	0
**ACEi (%)**	79	73
**ARB (%)**	11	9
**ARNi (%)**	21	36
**MRA (%)**	16	9
**Calcium Antagonist (%)**	11	27
**VKA (%)**	16	18

Values are expressed median [Q1;Q3]. BMI: Body mass index; bpm: beat per minute; LVEF: Left Ventricle Ejection Fraction; BBB: bundle branch block; QTc: QT interval corrected; COPD: Chronic obstructive pulmonary disease; SGLT2i : SGLT2 inhibitor; ACEi : Angiotensin-converting enzyme inhibitor; VKA: Vitamin K antagonist; SV: Stroke volume; TR vmax: Tricuspid regurgitation maximum velocity; LA diam : left atrial diameter.
